# Correction: Adult-born neurons modify excitatory synaptic transmission to existing neurons

**DOI:** 10.7554/eLife.83258

**Published:** 2022-09-08

**Authors:** Elena W Adlaf, Ryan J Vaden, Anastasia J Niver, Allison F Manuel, Vincent C Onyilo, Matheus T Araujo, Cristina V Dieni, Hai T Vo, Gwendalyn D King, Jacques I Wadiche, Linda Overstreet-Wadiche

**Keywords:** Mouse

Adlaf EW, Vaden RJ, Niver AJ, Manuel AF, Onyilo VC, Araujo MT, Dieni CV, Vo HT, King GD, Wadiche JI, Overstreet-Wadiche L. 2017. Adult-born neurons modify excitatory synaptic transmission to existing neurons. *eLife* **6**:e19886. doi: 10.7554/eLife.19886.

Published 30 January 2017

In the original article, there was an error in the Jackson Laboratory strain number specifying the *Pomc-Cre* mice used for experiments. In the methods section, it was incorrectly printed as Strain #005965 where it should have been Strain #010714. The authors apologize for this error that resulted from the availability of multiple lines of *Pomc-Cre* mice. This correction does not change the scientific conclusions.

The article has been corrected accordingly.

